# Two Brothers with Bardet-Biedl Syndrome Presenting with Chronic Renal Failure

**DOI:** 10.1155/2015/764973

**Published:** 2015-04-15

**Authors:** Cem Sahin, Bulent Huddam, Gulhan Akbaba, Hasan Tunca, Emine Koca, Mustafa Levent

**Affiliations:** ^1^Department of Internal Medicine, School of Medicine, Mugla Sıtkı Kocman University, Orhaniye Mahallesi İsmet Catak Caddesi, 48000 Mugla, Turkey; ^2^Department of Nephrology, Mugla Sitki Kocman University Education and Research Hospital, 48000 Mugla, Turkey; ^3^Department of Endocrinology, School of Medicine, Mugla Sıtkı Kocman University, 48000 Mugla, Turkey

## Abstract

Bardet-Biedl Syndrome (BBS) is a rarely seen autosomal recessive transfer disease characterised by retinal dystrophy, obesity, extremity deformities, mental retardation, and renal and genital system anomalies. BBS shows heterogenic transfer. To date, 18 genes (BBS1–18) and 7 BBS proteins have been defined as related to BBS. All of the defined BBS genes have been shown to be related to the biogenesis or function of cilia. Renal failure accompanying the syndrome, especially in the advanced stages, is the most common cause of mortality. Therefore, as one of the major diagnostic criteria, renal damage is of great importance in early diagnosis. This paper presents the cases of two brothers with BBS who presented with chronic renal failure.

## 1. Introduction

Bardet-Biedl syndrome (BBS) is a genetic syndrome with autosomal recessive transfer, characterised by retinal dystrophy, obesity, extremity deformities, mental retardation, and renal and genital system anomalies. Apart from these typical findings, various anomalies such as speech defects, dental anomalies, anosmia, cardiac anomalies, diabetes mellitus, hepatic fibrosis, anal agenesis, Hirschsprung disease, and neurological involvement may be seen together with the syndrome. Renal failure accompanying the syndrome, especially in the advanced stages, is the most common cause of mortality. Therefore, as one of the major diagnostic criteria, renal damage is of great importance in early diagnosis. In this report, the typical findings of the disease considered in two brothers with BBS who presented with chronic renal failure and the importance of family scanning are emphasised.

## 2. Case Presentations

### 2.1. Case  1

**Figure 1:**
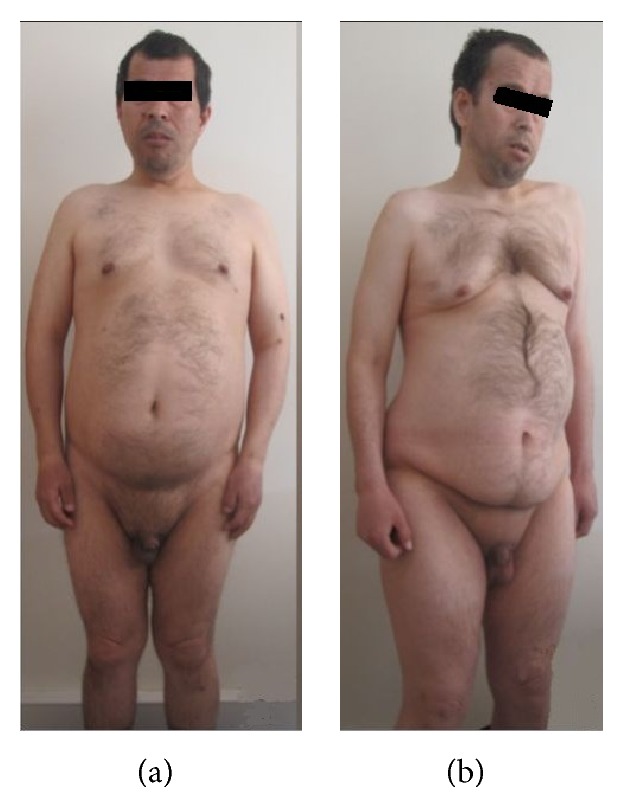
External appearance of the cases; (a) Case  1, (b) Case  2.

A 41-year-old male applied to the emergency department with complaints of diarrhea, nausea, and vomiting (Figure 1(a)). From the tests done, as the kidney function test results were high, he was admitted with an initial diagnosis of acute kidney failure. However, following appropriate fluid replacement therapy there was no improvement apart from a relatively small drop in the kidney function tests. On the urinary system USG, the right kidney was calculated as 100 × 40 mm with parenchyma thickness of 10 mm and the left kidney as 110 × 51 mm with parenchyma thickness of 13 mm and GFR as 84 mL/min. Serum creatinine level was 1.67 mg/dL. Other biochemical markers were normal. The case was accepted as stage II chronic kidney failure.

The patient had height of 159 cm, body weight of 72.5 kg, and BMI of 28.5 and vital signs were normal. The physical examination revealed mental retardation, speech defects, apathetic facial appearance, corneal matte appearance, strabismus, central obesity, micropenis, syndactyly, and polydactyly. The HOMA index was calculated as normal (2.18) for the patient who had central obesity. No pathology was observed from tests in respect of hormonal parameters.

**Figure 2:**
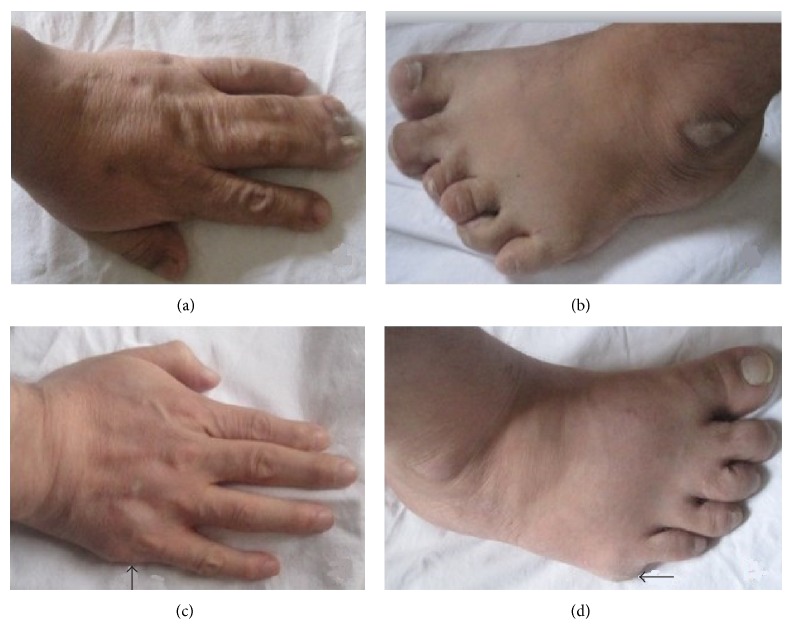
Extremities of the cases. (a) Polydactyly and syndactyly together in the left hand of Case  1, (b) polydactyly in the left foot of Case  1, and (c) and (d) right hand and foot of Case  2 (areas of previous surgery indicated by the arrow).

**Figure 3:**
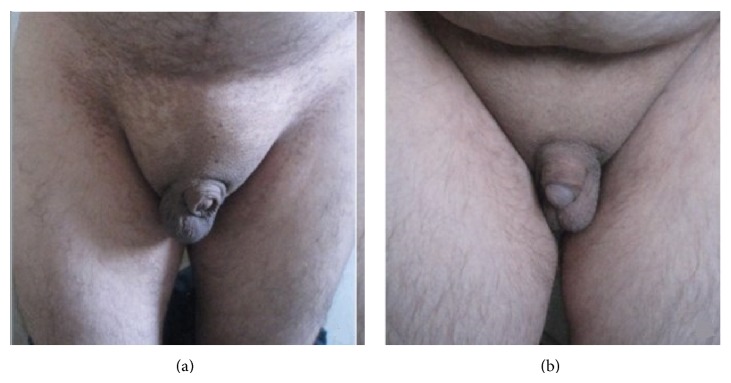
Hypogenitalism of the cases. (a) Micropenis and undescended left testis in Case  1. (b) Micropenis in Case  2.

In respect to cardiac pathology, left ventricle function was evaluated as normal on the echocardiography. A moderate level of mental retardation (IQ: 35–49) was determined in the psychiatric evaluation. There was polydactyly in both hands and feet and syndactyly of fingers 3–5 on the left hand (Figure 2). The patient had total loss of vision and, in the ophthalmological examination, exotropia and mature cataracts were observed in both eyes. Fundus examination could not be conducted because of the cataracts. Following ERG, cataract surgery was recommended to the patient. On scrotal USG, the left testis could not be visualized (intra-abdominal ectopy?). As the patient had a micropenis and undescended left testis (Figure 3(a)) in the sacral, pubic, and axillary hair areas, hormonal tests were performed and no findings of hypophyseal deficiency were observed. From all these findings, a diagnosis was made of BBS and renal failure. Questions were asked about similar anomalies in the family and the brother was called to the polyclinic for testing.

### 2.2. Case 2

The 38-year-old male had a height of 165 cm, a weight of 70 kg, and BMI of 25.7 and vital signs were normal (Figure 1(b)). The patient had mental retardation from birth and the physical examination determined speech defects, strabismus, loss of vision, gynecomastia, central obesity, and micropenis. The patient history revealed 6 fingers on both hands and feet from birth and the extra fingers had been surgically removed for cosmetic reasons during childhood.

In the hormonal tests done because of hypogenitalism (micropenis) (Figure 3(b)) in the sacral, pubic, and axillary hair areas, no findings of hypophyseal deficiency were observed. In the echocardiography applied in respect to cardiac pathology, the left ventricle function was evaluated as normal. Moderate level mental retardation (IQ, 35–49) was observed in the psychiatric evaluation and in the fundus of both eyes, and widespread bone spicules were determined. With these findings, the patient was diagnosed with retinitis pigmentosa and ERG was recommended. In kidney function tests applied 6 months previously, creatinine level had been observed to be high (4.98 mg/dL), but the patient had not been followed up by either the Nephrology or the Internal Medicine Department. On urinary USG, the right kidney was observed to be 92 mm in size with a parenchyma thickness of 6 mm and the left kidney was 76 mm in size with a parenchyma thickness of 6 mm. In addition, a 5 mm diameter stone was observed in the mid-section of the left kidney and simple cysts of maximum 10 mm diameter in both kidneys. With these findings and GFR calculated at 13 mL/min, the patient was diagnosed with BBS and end-stage kidney failure. The patient was referred to the Nephrology Department for renal replacement treatment.

## 3. Discussion

In Bardet-Biedl syndrome (BBS), apart from the major findings, different clinical effects can be observed evaluated under the heading of minor criteria, such as speech defects, dental anomalies, anosmia, ataxia, diabetes mellitus, and cardiac anomalies. Rod-cone dystrophy (90–100%), obesity (72–92%), polydactyly (63–81%), genital anomalies (59–98%), learning difficulties (50–61%), and renal anomalies (20–53%) are the major components of BBS. Speech disorders (54–81%), developmental delay (50–91%), diabetes mellitus (6–48%), dental anomalies (51%), congenital heart disease (7%), brachydactyly/syndactyly (46–100%), ataxia/poor coordination (40–86%), cardiopathy (10%), deafness (11–12%), and anosmia/hyposmia (60%) are the minor components of BBS [1]. According to this, at least 4 major criteria or 3 major and 2 minor criteria together are sufficient for a diagnosis [1]. BBS, which is evaluated among the rarely seen genetic transfer diseases, shows autosomal recessive hereditary properties. While it is seen more often in isolated communities or those where there is widespread consanguinity (Kuwait 1 : 17,000 and Newfoundland 1 : 18,000), incidence in Europe and North America has been reported as 1 : 140,000–160,000 [1]. BBS shows a heterogeneous genetic transfer property. The latest update in 2014 related to the genetic characteristics of the syndrome reported that to date a relationship has been established between BBS and 18 genes (BBS 1–18) and 7 proteins (BBS 1, 2, 4, 5, 7, 8, and 9). Although the BBS mutation spectrum shows differences between populations, approximately 70–80% of the cases have been determined with BBS gene mutation [2]. Even though the results of some studies have reported a mild phenotypic correlation with BBS gene mutation, a clear and definitive relationship has not been shown between genotype and phenotype in BBS [3] and the correlation between the BBS genotype and phenotype varies among and within families. In the study of Shin et al., it was a genetically confirmed BBS case in a Korean family with a compound heterozygous mutation of the BBS7 gene [4].

While the underlying pathology of BBS clinical findings remains unknown, the study results of several subjects have suggested that the basic reason for this pleiotropic disease originates from basal body or cilia dysfunction [5]. Cilia are organelles resembling hairs which are found in nearly all cells of the body. Cilia are classified into two groups as motile or nonmotile (primary). Nonmotile cilia are thought to function as a sensory organelle in the regulation of signal transduction pathways. These primary cilia, which appear on the apical cell surface, mediate the transmission of mechanical and chemical stimuli through different signalling pathways [6]. Previous studies have shown a relationship of all known BBS genes with cilia biogenesis or function [7]. The defect occurring in nonmotile cilia has been shown to be clinically related to retinitis pigmentosa, polydactyly, situs inversus, learning difficulties, and cystic diseases of the kidney, liver, and pancreas [8].

BBS clinical characteristics generally start to be seen slowly in the first decade of life. Most cases are diagnosed in late childhood or early adolescence. Extremity anomalies determined at birth are one of the most important clinical signs for diagnosis. In terms of indicating diagnosis, extremity or renal anomalies at birth or in the intrauterine period are important. However, the variability which can be seen in these two characteristics and the late onset of other indications may be the reason for the diagnosis of the cases after childhood.

BBS is one of the most important causes of syndromic retinal dystrophy, which is seen with severe sight problems during preadolescence and with generally total loss of vision before the second decade [7]. Ophthalmological findings in BBS patients may be seen in various forms from iris coloboma, bilateral aniridia, cataracts, myopia, to anophthalmia. The classic form is retinitis pigmentosa. Different from classic retinitis pigmentosa which is not related to systemic diseases, in these patients macular degenerative changes are seen in the early stages and, in most cases, total loss of vision develops before the second decade. Different forms of retinal dystrophy have been defined, such as cone-rod dystrophy, rod-cone dystrophy, choroidal dystrophy, and global severe retinal dystrophy. Cone-rod dystrophy is known as progressive retinal degeneration and is characterised by reduced visual acuity and impaired colour vision. Ophthalmological findings are observed in 90–100% of BBS cases [7].

In BBS cases, obesity, especially central obesity, is the second major clinical finding in terms of significance and prevalence. Rates of obesity incidence have been reported as 72–92% in BBS cases. While birthweight is generally normal, 1 in 3 syndromic babies with normal birthweight becomes obese before the age of 1 year [9]. Obesity starts in early childhood and continues to increase over time. In some BBS cases, type II DM may develop related to the degree of obesity.

Extremity anomalies such as polydactyly and syndactyly as the earliest seen signs in BBS patients which are determined at birth are one of the most important clinical indicators for diagnosis. Typically, polydactyly is seen in 63–81% of BBS cases. As it is seen from birth onwards, it is the most important finding for suspicion of the syndrome [8]. Most commonly, postaxial polydactyly, polydactyly, and brachydactyly are seen in both hands and feet. Other extremity anomalies are often reported such as brachydactyly and syndactyly in both hands and feet.

Hypogonadism as another major criterion of the syndrome may be seen with delayed onset of puberty in both genders, hypogenitalism in males and genital anomalies in females. Gonadal dysfunction is seen more often in male patients than female patients. Small penis size and decreased testis volume are often seen in male patients and in female patients; there is often a delay in the onset of the menstrual cycle. In female patients, hypoplasia of the fallopian tubes, uterus, and ovaries, vaginal atresia, and genital malformations such as hematocolpos and vesicovaginal fistula may be seen.

Mental retardation, learning difficulties, speech defects, autism, and behavioural problems such as psychosis are neuropsychiatric impairments seen in BBS. Mental retardation is the fourth most important characteristic of the syndrome. Psychosocial development is retarded and IQ test scores are low. Cognitive dysfunction is noticed in most cases after they start school. It has been shown that primary cilia are one of the most important organelles in the human brain and are necessary for the hippocampal development stages. It has been shown in a study that the hippocampus volume of BBS cases is low in comparison with healthy individuals [10].

Urogenital system malformations are common in BBS and all cases should be routinely investigated for renal anomalies. The onset of renal dysfunction has not been evaluated as a major component of the syndrome. However, in the subsequent period, the observation of renal involvement in most cases and that it is the most common cause of mortality causes renal dysfunction to be defined as a major criterion of the syndrome [11].

Although a range of renal anomalies may be seen, the classic appearance is tubular disease and anatomic malformations. Renal symptoms are generally nonspecific. In approximately a third of cases, polyuria/polydipsia is seen to be associated with defective vasopressin-resistant urine concentration [9]. In patients who have not developed polyuria or where it has been ignored if present, the first renal sign may be chronic kidney disease or end-stage kidney disease [12].

The most commonly seen renal function impairment is impaired urine concentration capacity. In most cases of the urine concentration defect, no deterioration in renal function tests is observed [9, 13]. Hypertension may be seen in the early stages of life; two-thirds of all patients have hypertension with 30–50% of the patients aged below 34 years [14]. Hypertension in the absence of renal failure is not a commonly seen characteristic.

In kidney histology, chronic interstitial nephritis, mesangial proliferative glomerulopathy, and structural changes in the glomerular basal membrane may be observed. In some cases, advanced renal failure may develop due to cystic kidney disease. This may cause recurrent urinary system infections, chronic pyelonephritis, and kidney failure. As radiological findings in addition to renal function impairments in BBS patients, structural renal defects may be seen such as renal cysts, diverticulae, calyceal deformity, fetal lobulation, scars, and diffuse cortical loss.

An effective multidisciplinary approach is required to manage this pleiotropic situation. There is no definitive treatment method for BBS. Complications related to BBS should be treated symptomatically. There should be an awareness of complications for which BBS has laid the base and patients should be followed up in this respect. Effective weight management is necessary to prevent related morbidities such as metabolic syndrome. Patients with metabolic syndrome should be evaluated in respect to potential hypertension, diabetes mellitus, hyperlipidemia, and cardiovascular diseases. A detailed ophthalmological evaluation including electroretinogram (ERG) is required to determine the onset and extent of retinal dystrophy. Renal ultrasound should be applied to all patients at the time of diagnosis to discount renal malformation. Patients with findings of chronic renal failure should be referred to a nephrologist. Developmental and educational evaluations are necessary for all patients [8].

BBS generally shows autosomal recessive inheritance. Although triallelic inheritance has been reported in some cases, it is difficult to determine as it is seen in less than 10% of all cases. Information should be given about the heterogeneous nature of the patients and their relatives. In families who know the mutation causing the disease, there is the possibility of preimplantation genetic diagnosis or prenatal testing. In families at risk whom the mutation is unknown to, ultrasonography can be applied in the third trimester for the visualization of axial polydactyly and renal malformations [8].

## 4. Conclusion

For patients presenting with impaired kidney function accompanied by findings such as polydactyly, mental retardation, obesity, vision problems, or micropenis, BBS should certainly be considered.

However, the syndrome is rarely observed; kidney involvement is common.

When it is considered that renal failure is the most common cause of mortality, early diagnosis of renal damage is of great importance.

The syndrome and renal damage can be determined in early stages by family scanning.
